# Differential Dynamics of *CALR* Mutant Allele Burden in Myeloproliferative Neoplasms during Interferon Alfa Treatment

**DOI:** 10.1371/journal.pone.0165336

**Published:** 2016-10-20

**Authors:** Lasse Kjær, Sabrina Cordua, Morten O. Holmström, Mads Thomassen, Torben A Kruse, Niels Pallisgaard, Thomas S. Larsen, Karin de Stricker, Vibe Skov, Hans C. Hasselbalch

**Affiliations:** 1 Department of Haematology, Zealand University Hospital, Roskilde, Denmark; 2 Department of Clinical Genetics, Odense University Hospital, Odense, Denmark; 3 Department of Surgical Pathology, Zealand University Hospital, Roskilde, Denmark; 4 Department of Haematology, Odense University Hospital, Odense, Denmark; 5 Department of Pathology, Odense University Hospital, Odense, Denmark; Queen's University Belfast, UNITED KINGDOM

## Abstract

Discovery of somatic mutations in the calreticulin gene (*CALR*) has identified a subgroup of Philadelphia-negative chronic myeloproliferative neoplasms (MPN) with separate haematological characteristics and prognosis. *CALR* mutations serve as novel markers both of diagnostic value and as targets for monitoring molecular responses during therapy. Interferon-α (IFN) selectively targets the malignant clone in a subset of MPN patients and can induce both haematological and molecular remissions in *CALR* mutated essential thrombocythemia (ET) patients. We investigated the response to IFN in a cohort of 21 *CALR* mutated MPN patients including ET, prefibrotic primary myelofibrosis (pre-PMF), and primary myelofibrosis (PMF) with a median follow-up of 31 months. For evaluation of a molecular response, we developed highly sensitive quantitative PCR (qPCR) assays for monitoring the mutant allele burden of the two most prevalent *CALR* mutations (type 1 and type 2). Thirteen patients (62%) experienced a decrease in the mutant allele burden with a median decline of 29% from baseline. However, only four patients, including patients with ET, pre-PMF, and PMF diagnosis, achieved molecular responder (MR) status with >50% reduction in mutant allele burden according to European LeukemiaNet (ELN) guidelines. MR patients displayed significant differences in the dynamics of the *CALR* mutant load with regard to time to response and dynamics in mutant allele burden after discontinuation of IFN treatment. Furthermore, we highlight the prognostic value of the *CALR* mutant allele burden by showing a close association with leucocyte- and platelet counts, hemoglobin concentration, in addition to plasma lactate dehydrogenase (LDH) irrespective of molecular response and treatment status.

## Introduction

The Philadelphia-chromosome negative classical chronic myeloproliferative neoplasms (MPN) include essential thrombocythemia (ET), polycythaemia vera (PV), primary myelofibrosis (PMF), and prefibrotic/early PMF (pre-PMF). These phenotypically different entities are clonal haematopoietic stem cell malignancies characterised by de-regulated myeloproliferation and accumulation of myeloid cells. The discovery of mutations in the calreticulin (*CALR)* gene in patients with MPNs has been the most important finding of genetic aberrations with phenotypic impact since the *JAK2-*V617F mutation, increasing the diagnostic accuracy for these neoplasms considerably [1–3]. The *CALR* mutations are present almost exclusively in *JAK2-*V617F negative patients with ET and PMF [2–6], although cases of refractory anaemia with ring sideroblasts associated with thrombocytosis (RARS-T) and PV [7, 8], in addition to rare cases of *JAK2-*V617F or BCRABL1 co-positivity have been reported [9, 10]. More than 50 different mutations consisting of deletions, insertions or a combination thereof map to exon 9 of the *CALR* gene and result in a +1 frameshift of the coding sequence introducing a novel c-terminus, which has very recently been shown to hold the MPL-dependent oncogenic properties [11, 12]. The majority of mutations consists of a 52 base pair (bp) deletion (type 1) and a 5 bp insertion (type 2). Type 2 and type 2-like mutations are more frequent in ET patients and associated with a lower risk of thrombosis [13] whereas the type 1 and type 1-like mutations are associated with myelofibrotic transformation and are more frequent in PMF patients [5, 13]. So far, analysis of relatively small patient cohorts has led to conflicting results regarding the prognostic impact of type 1 and type 1-like mutations [14–16].

Treatment with interferon-α (IFN) effectively controls the excess myeloproliferation. Besides normalisation of haematological parameters, IFN has the potential to induce deep molecular responses in both *JAK2-*V617F positive MPN patients [17–21] and in a subset of patients with *CALR* mutated ET patients [22, 23]. Minimal residual disease with very low *JAK2*-V617F allele burden and normal bone marrow has been described during long-term IFN-treatment of early stage disease (ET and PV). Since the *JAK2*-V617F mutation per se induces genomic instability and is considered as a driver of chronic inflammation by inducing reactive oxygen species (ROS) [24–26], IFN-treatment is considered a highly rational approach targeting the malignant clone. Furthermore, by reducing the JAK2-*V617F* allele burden and thereby the production of ROS IFN also disrupts chronic inflammation that in a self-perpetuating vicious cycle fuels expansion of the MPN-clone [25, 26].

For routine diagnostic purposes, screening for *CALR* mutations by fragment analysis is sufficiently sensitive as the majority of *CALR* mutation positive samples appear to have above 15% mutant alleles [27]. However, post-transplantation relapse monitoring of *CALR* mutation positive AML patients and evaluation of treatment response in ET patients during IFN treatment have established the value of sensitive and quantitative determination of *CALR* mutations [22, 23, 28, 29]. Sensitive quantitative polymerase chain reaction (qPCR) assays are currently the method of choice for monitoring mutant allele burden including deep IFN-induced remissions as seen in a subset of *JAK2-*V617F positive MPN patients [17]. Consensus reports issued by the European LeukemiaNet (ELN) have addressed specific recommendations regarding *JAK2-*V617F monitoring [30, 31].

In the current study, we developed reliable and highly sensitive qPCR assays for monitoring the allele burden of *CALR* type 1 and type 2 mutations. We investigated the dynamics of molecular responses in patients with ET, pre-PMF, and PMF during and after IFN treatment and analysed the association with haematological parameters to estimate how well the *CALR* mutant allele burden reflects the disease phenotype and disease control.

## Materials and Methods

### DNA extraction and mutation analysis

DNA was extracted from peripheral blood on a Qiasymphony DSP using the DSP DNA Mini Kit (Qiagen, Hilden, Germany) according to the manufacturer’s instructions. As a part of the routine diagnostic work up, the patients were analysed for the *JAK2*-V617F mutation by the Larsen assay [31] and initial identification of *CALR* mutations was performed by fragment analysis as previously described [2]. GeneMarker (Softgenetics, PA, USA) was used for fragment analysis and assessment of peak heights for quantitative analysis.

### QPCR for CALR mutant allele burden

We designed qPCR assays targeting the *CALR* type 1 and type 2 mutations. The sequence of the DNA in the region with the *CALR* mutations contains several simple and tandem repeats posing a challenge for the design of specific primers as the specificities of the wildtype (wt) and type 1 specific primers for their respective targets determine the sensitivity of the assay. The 52 bp region, which is deleted for type 1 mutation, is flanked by two identical 7 bp repeats in the wt sequence and one of these remains after the deletion. The specificity of the primer is thus dependent on differences between the area 5’ to the second 7 bp repeat in the wt sequence and the 5’ area of the 7 bp region in the mutated sequence. The first 15 bases 5’ to the 7 bp repeat contain 11 mismatches between wt and the type 1 sequence and eight of these are weak mismatches (S1 Fig) [32, 33]. To achieve maximum specificity the 3’ of the forward primer was placed in the 7 bp sequence and to increase the specificity of the assays a 3’ intended mismatch was introduced into the design and are indicated by bases in lower case in the forward primers.

Taqman assays specific for both type 1 and type 2 mutations were designed to determine the mutant allele burdens using a wildtype (wt) or mutation specific forward primer, a common probe and a common reverse primer. The reaction was carried out on 100 ng genomic DNA in a volume of 25 μl using 200 nM probe and 300 nM primers with Applied Biosystems^™^ TaqMan^™^ Universal Master Mix II with uracil-N-glycosylase (UNG) (Life technologies, Paisley, UK). PCR amplification conditions were an initial 2 minutes 50°C step for UNG activity, followed by a 10 minutes 95°C activation of the polymerase, and then 50 cycles of 15 seconds at 95°C followed by 60 seconds at 60°C. The reactions were performed in triplicates on a Quantstudio (Life technologies, Paisley, UK). The *CALR* type 1 and type 2 assays were designed with a common reverse primer 5’-GCCTCTCTACAGCTCGTCCTTG-3’ and a common reverse probe 6-FAM-CCGGGGACATCTTCCTCCTCATCT-TAMRA. The forward primers for the type 1 assay were a wildtype specific primer 5’-CAGGACGAGGAGCAGAGaCT-3’ and a mutation specific primer 5’-ACAGGACGAGGAGCAGAGaAC-3’. The forward primers for the *CALR* type 2 assay were a wildtype specific primer 5’-GAGGAGGAGGCAGAGGACAtGG-3’, and a mutation specific primer 5’-GGAGGAGGAGGCAGAGGACAtTT-3’. All primers have an intended mismatch at the 3’-minus 2 position. PCR efficiencies were determined by several rounds of standard curves generated from 5-fold dilutions of type 1 and type 2 mutation-positive patient DNA. Mutant allele burdens were calculated according to the formula (10^((Cqmut − Yintercept mut)/ ((Cqmut − Yintercept mut) + (Cqwt − Yintercept wt))^) x 100%. For routine sensitivity, we defined a cut-off limit 10 fold higher than the specificity resulting in <0.01% for the type 1 and 0.02–0.04% for the type 2 assay.

### Calibration of qPCR using droplet digital PCR

To determine Y-intercepts and slopes of the standard curves for qPCR, the numbers of wt and mutated copies were assessed by droplet digital PCR (ddPCR). The reactions were performed on 5 fold dilutions of 80 ng of genomic DNA. The ddPCR was performed on a QX-100 Droplet Digital System (Bio-Rad, Hercules, CA), and the mutated allele burden was analysed by multiplex PCR assays for the *CALR* type 1 and type 2 mutations on 10–17.000 droplets. The assays and reactions were performed as previously described [29], with the following changes for the type 2 assay: primer concentrations were 300 nM and probe concentration was 200 nM while the annealing temperature was set to 60°C for both assays.

### Patients and clinical data

We included all IFN-treated *CALR* positive MPN-patients from our centre with a minimum of six months IFN treatment and a minimum of two *CALR* measurements during treatment. The patients were diagnosed according to the existing WHO criteria. Indications for IFN treatment were elevated platelet- and/or leucocyte counts, resistance, or adverse effects to prior cytoreductive treatment. Neither prior treatment with other cytoreductive drugs nor discontinuation of IFN treatment were exclusion criteria. Clinical data were collected retrospectively from medical records, and follow up time was defined as the time from initiation of IFN therapy until date of the last sampling for *CALR* qPCR analysis.

Since spleen size was not assessed in all patients at all time points, at which haematological and molecular analyses were done only haematological and not clinico-haematological responses were assessed.

For patients with ET and pre-PMF, we defined a complete haematological response as a leucocyte cell count <10 x 10^9^/L and platelet count <400 x 10^9^/L lasting at least 12 consecutive weeks [34]. For those not fulfilling these criteria, a partial haematological response was obtained by a platelet count <600 x 10^9^/L or a reduction of >50% from baseline [34]. For PMF, we defined a sustained complete haematological response as having a haemoglobin concentration within the range 10 g/dL < x < upper normal limit (male: 16.9 g/dL, female: 15.3 g/dL), platelet count within 100 x 10^9^/L < x < 400 x 10^9^/L, and neutrophil cell count within 1 x 10^9^/l < x < 7 x 10^9^/L lasting at least 12 consecutive weeks [35].

For evaluation of molecular response, we defined it as the difference between the *CALR* allele burden closest to initiation of IFN treatment and the latest *CALR* allele burden measurement during treatment. For ET and pre-PMF, we adapted and defined a *CALR* mutant allele burden response in accordance with the ELN definition from 2009 [34] regarding the *JAK2-*V617F mutation response: for patients with an initial allele burden of >50%, a partial molecular response was defined as a minimum reduction of 25% from the initial measurement, whereas for patients with an initial allele burden of 10–50%, a partial response required a reduction of at least 50% [34]. For PMF, we used the definition on molecular remission from the International Working Group-Myeloproliferative Neoplasms Research and Treatment (IWG-MRT) and ELN consensus report: for patients with an initial allele burden of at least 20%, a partial response was obtained when a minimum 50% reduction in allele burden was observed [35].

### IFN treatment

The prescribed IFN formulation was predominantly subcutaneous injections of pegylated IFN -2a (Pegasys^®^ with median dose: 45 microgram (μg) per week (180 μg/month), range 11–135 μg per week). A subset of patients was treated with pegylated IFN -2b (PegIntron^®^ with doses 25–90 μg per week) or human leucocyte IFN (Multiferon^®^ 3 x 3 million IU per week). Other cytoreductive treatment included hydroxyurea, anagrelide, busulfan, JAK2 inhibitor.

### Approvals

The study was approved by the Regional Committee on Health Research Ethics and the Danish Data Protection Agency.

### Statistics

We generated graphs and statistics using Graphpad Prism and Stata/SE 14.0. In order to include all paired measurements of blood counts and mutant *CALR* allele burden in each patient when analysing the association between the allele burden and haemoglobin, platelet count, leucocyte count, and plasma lactate dehydrogenase (LDH), respectively, we applied a mixed effect model using restricted maximum likelihood and Kenward-Roger test. Fishers exact test was used to compare frequencies between groups, and the Mann-Whitney test (or Kruskal Wallis test when analysing more than two groups) was used for unpaired quantitative variables. The Wilcoxon matched-pairs signed-ranks test was used for comparing the initial *CALR* level with the level of the latest measurements.

## Results

### Assays for quantifying CALR type 1 and type 2 mutations

**Fig 1:**
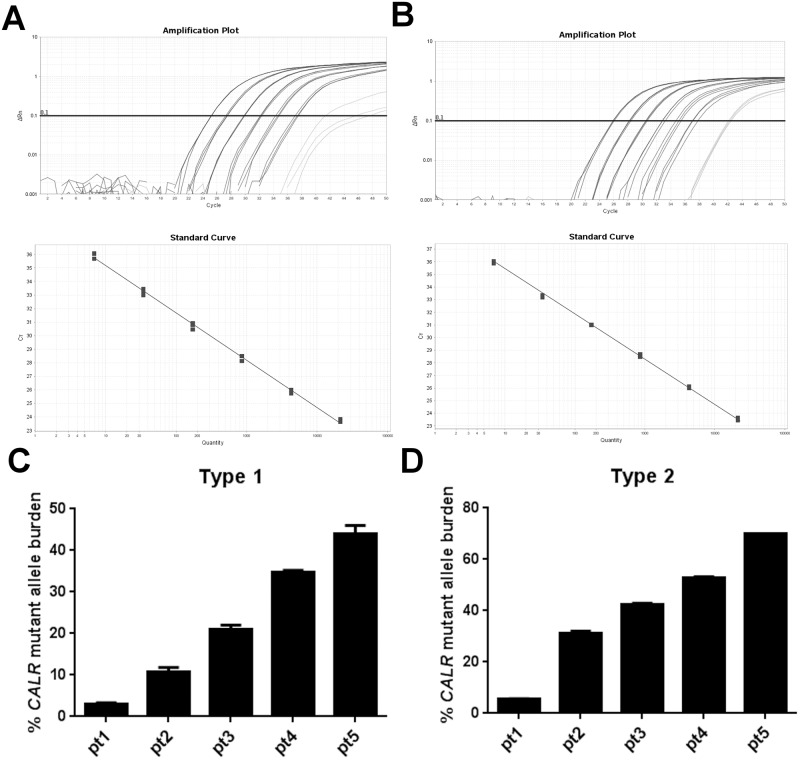
Performance of *CALR* qPCR assays for type 1 and type 2 mutations. Logarithmic amplification curves, standard curves and measurements of assay reproducibility of (A) type 1 and (B) type 2 mutations. The qPCR amplification and standard curves were generated from 5-fold dilutions of mutated patient DNA in wildtype (wt) DNA. The amplification curves appearing at Cq values above 41 are unspecific amplification of wt DNA. Reproducibility of the type 1 and type 2 assays was evaluated by performing three independent runs on five patient samples with the *CALR* type 1 mutation and five patient samples with the *CALR* type 2 mutation with varying amounts of mutated alleles demonstrating robust performance suitable for clinical purposes.

We designed qPCR assays targeting the *CALR* type 1 and type 2 mutations. Standard curves were generated using material, where the exact copy number was determined by ddPCR to ensure correct quantification. The qPCR standard curves demonstrated correlation coefficients above 0.990 for all curves with slopes varying from -3.3 to -3.4 and Y-intercepts varying from 39.3 to 40.5 (Fig 1A and 1B).

To ensure that the reproducibility of the assays was of sufficient quality for quantifying clinical samples, five patients with either type 1 or type 2 mutations in varying amounts of mutated alleles were analysed on three separate occasions. Both assays performed exceptionally well, with the type 1 assay displaying coefficients of variation in the range of 1.7% - 11% for mutant allele burdens in the range of 2.8% - 46% (Fig 1C) and the type 2 assay displaying coefficients of variation in the range of 0.0% - 3.2% for mutant allele burdens in the range of 5.5% - 70% (Fig 1D). Unspecific amplification of the wt template by the mutant specific primers for the two assays usually had a ΔCq of 16–17 and the specificities were thus below the theoretical possible detection limit for 100 ng DNA (1 in 30,000 copies). Routine sensitivity was set to be 3.3 log or 10 fold higher than the specificity (usually 0.01–0.02% for 100 ng DNA). In two patients with non-type 1 and 2 mutations, we estimated the mutant allele burden pseudo-quantitatively by measuring the peak heights from the fragment analysis.

Preferential amplification of shorter amplicons may lead to over- or underestimation of the mutant allele burden when using peak heights obtained in fragment analysis for quantitative assessment of the mutant allele burden. To determine the accuracy of using peak heights from fragment analysis for quantitative determination of type 1 and type 2 mutations, we compared mutant allele burden estimations based on either fragment analysis or ddPCR calibrated qPCR in the same patients. Using fragment analysis for determination of mutant allele burden resulted in a consistent overestimation of the type 1 mutant allele burden (mean relative difference: 66%, 95% CI:[63–69%], n = 109), and a fairly accurate estimation of the type 2 mutant allele burden when compared to qPCR (mean relative difference: -2%, 95% CI:[-3;-4%], n = 47).

### Demographics and clinical data

Twenty-one patients with *JAK2-*V617F negative, *CALR* -mutated MPN met the inclusion criteria and encompassed 5 patients with ET, 4 patients with pre-PMF, and 12 patients with PMF. The cohort consisted of 9 males and 12 females with a median age at the time of diagnosis of 48 years (range 30–73 years). At the time of diagnosis, 7 out of 19 (37%) patients suffered from anaemia, 16 of 19 (84%) patients had thrombocytosis, 8 of 19 (42%) patients suffered from leucocytosis, and 16 of 18 (89%) patients displayed elevated LDH (Table 1). Twelve patients had splenomegaly; in 7 patients determined by sonography with a median spleen size of 15 cm (range 13.5–20 cm) and in 5 patients determined by computed tomography (CT) with a median size of 15 cm (range 14–25 cm).

**Table 1:** Demographics and clinical baseline characteristics for molecular responders versus molecular non-responders and total cohort.

	MR	Non-MR	P	Entire cohort
**Number of patients**	4	17		21
**Age at diagnosis, years**	52 (34–60)	49 (30–73)	0.89	49 (30–73)
**Sex ratio, M/F**	1/3	9/8	0.59	10/11
**Diagnosis**			1.00	
- **ET**	2 (50%)	10 (59%)		12 (57%)
- **pre-PMF**	1 (25%)	4 (24%)		5 (24%)
- **PMF**	1 (25%)	3 (18%)		4 (19%)
**Type of calreticulin mutation**			0.70	
- **type 1**	4 (100%)	11 (65%)		15 (71%)
- **type 2**	0	4 (24%)		4 (19%)
- other*	0	2 (12%)		2 (10%)
**At diagnosis**†				
- **platelet count, x 10^9^/L**	790 (733–809)	712 (138–1470)	0.23	733 (138–1470)
- **leucocyte count, x 10^9^/L**	7.4 (5.8–18.9)	8.8 (5.4–14.4)	0.62	8.7 (5.4–18.9)
- **haemoglobin, g/dL**	13.2 (12.1–14.2)	12.6 (7.9–14.3)	0.29	12.7 (7.9–14.3)
- **LDH, U/L**	276 (234–356)	376 (158–624)	0.20	337 (158–624)
- **palpable splenomegaly**	0	3 (18%)	0.51	3 (14%)
**IFN treatment, months**	31 (6–87)	31 (7–137)	0.93	31 (6–137)

MR, molecular responders; non-MR, molecular non-responders; ET, essential thrombocythemia; pre-PMF, prefibrotic primary myelofibrosis; PMF, primary myelofibrosis; LDH, lactate dehydrogenase; IFN, interferon.

Numbers are given as median (range) or number (%), and P values for differences between MR versus non-MR are shown.

* 1191-1142del and 1102-1136del34

^†^ missing values on 2 patients.

**Fig 2:**
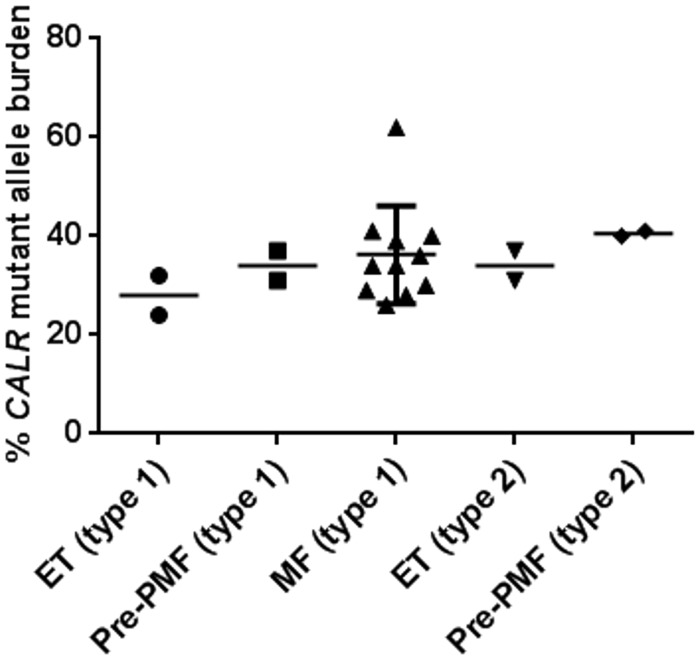
Distributions of initial *CALR* mutant allele burdens according to diagnosis and mutation type. Mutant allele burdens shown for type 1 ET (n = 2), type 1 pre-PMF (n = 2), type 1 PMF (n = 11), type 2 ET (n = 2), and type 2 pre-PMF (n = 2) patients. The mean is indicated by the horizontal line, and the type 1 PMF group is depicted with mean ±SD.

Of the 21 *CALR* mutated patients, 15 (71%) carried type 1 mutations, 4 (19%) type 2 mutations, 1 (5%) a non-type1 52 bp deletion shifted 1 bp upstream (1098-1149del), and 1 (5%) had a 34 bp deletion (1102-1135del34, type 4). The initial mutant allele burden for type 1 mutations (median: 34%, range: 21–62%, n = 15) resembled that for type 2 mutations (median: 39%, range: 31–41%, n = 4) and we observed a slight tendency to an increase of the mutant allele burden from ET to PMF for both groups (Fig 2). At the time of data collection, 16 patients (76%) still received IFN, whereas treatment had been discontinued in five patients (24%) because of side effects (thyroid dysfunction, abnormal liver function tests, leucopenia or mood changes/depression). Twelve patients (57%) had also received other cytoreductive treatment.

The median time from diagnosis to last *CALR* qPCR measurement was 60 months (range 12–385 months), the median duration of IFN treatment was 31 months (range 6–137 months), and the median follow-up from start of treatment to last *CALR* qPCR measurement was 31 months (range 7–137 months) (Table 1).

### Haematological -and molecular response during IFN treatment

Fourteen (82%) of the 17 patients with elevated blood cell counts at start of IFN treatment obtained a complete haematological remission during IFN treatment after a median of 10 weeks (range: 3–70 weeks). Among the remaining three patients, two obtained a partial haematological response (1 ET and 1 pre-PMF), and one had a haematological response but for less than 12 consecutive weeks (PMF). Two patients normalised their spleen size on a follow-up scan during treatment (follow-up scan was performed in 9 of the 12 patients).

**Fig 3:**
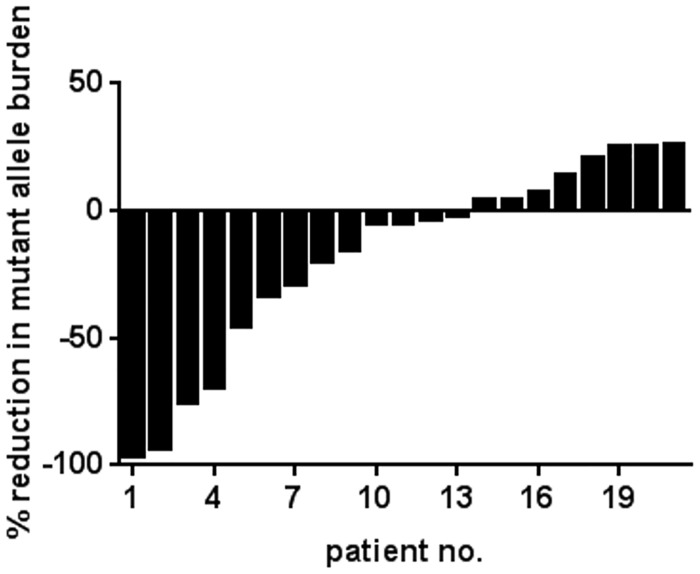
Allele burden response to IFN in *CALR* mutated MPN patients. Waterfall plot visualising percentage changes in the mutant allele burden of *CALR* from baseline (start of IFN treatment) to the latest *CALR* qPCR measurement in 21 patients with a median follow-up of 31 months (range 7–137 months).

Waterfall plot visualisation of the individual changes in *CALR* mutant allele burden revealed that 13 of the 21 patients (62%) experienced a decrease in mutant allele burden with a median reduction of 29% (range: 2–96%) and 8 (38%) demonstrated an increase with a median of 18% (range: 4–26%) (Fig 3).

According to the ELN guidelines, 4 (19%) of the 21 patients achieved a partial molecular response. These included an ET patient, a pre-PMF patient and two PMF patients that all had type 1 mutations. The median mutant allele burden at start of treatment for the two groups was MR patients: 30% (range: 24–62%, n = 4) and molecular non-responder (non-MR) patients: 36% (range: 21–46%, n = 17). A pre-PMF patient with type 2 mutation and a PMF patient with a type 1 mutation had reductions in the mutant allele burden of 49% and 45%, respectively but were considered to be non-MRs according to the ELN guidelines. No significant differences were identified when comparing the baseline data of MR- versus non-MR patients (Table 1).

All MR patients obtained a complete haematological response within a median time of 13 weeks (range: 4–70 weeks) and all normalised their LDH within 31 weeks. Two had discontinued IFN treatment at the time of data collection, one because of hypothyroidism and the other because of leucopenia. After 13 and 22 months of discontinuation, respectively, they both still maintained a complete haematological response and normal LDH without any cytoreductive treatment.

In the non-MR group, 10 out of 13 (77%) patients with elevated blood cell counts at IFN initiation obtained a complete haematological response (median time: 10 weeks, range 3–28 weeks) with only 5 (50%) patients also obtaining normalisation of LDH with a median time of 23 weeks (range 2–181 weeks). Three of the non-MR patients had discontinued IFN treatment at the time of data collection.

Interestingly, no anaemia or marked bone marrow fibrosis (grade III—IV) was observed in the MR patients at the time of diagnosis, whereas this was present in 7 (data available in 15 patients) (47%) and 6 (data available in 16 patients) (38%) of the non-MR patients, respectively. In addition, we noticed that 4 (24%) of the non-MR patients had experienced second cancer (not including basal cell carcinoma) compared to none in the MR patients.

### Differential dynamics of the CALR mutant allele burden

**Fig 4:**
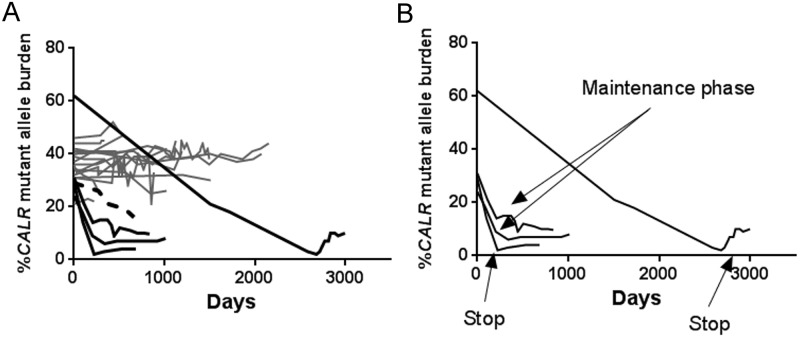
Dynamics of the *CALR* mutant allele burden during IFN treatment. A) Dynamics of allele burden in patients from start of IFN treatment with black lines indicating molecular responder (MR) patients and grey lines indicating molecular non-responder (non-MR) patients. The dashed black line denotes a non-MR exhibiting MR dynamics, but with an insufficient reduction in mutant allele burden for obtaining MR status (45% reduction). B) Mutant allele burdens over time for MR patients with indications of reduction of IFN dose (maintenance phase) and IFN withdrawal (stop).

The four MR patients demonstrated a median decrease in mutant allele burden of 84% (range 69% - 96%). Three had initial mutant allele burdens of 28%, 24%, and 31% and achieved molecular responses after 116, 207 and 307 days, respectively. The fourth patient with an initial mutant allele burden of 60% exhibited a much slower decrease rate and did not achieve molecular response until 679 days after starting IFN treatment with a 50% reduction after 1359 days of treatment with IFN. The mutant allele burden continued a steady decrease until the end of IFN treatment more than 93 months later (Fig 4A and 4B).

The two MR patients that terminated IFN treatment demonstrated either slow or a fast response during treatment (Fig 4B). We observed a faster molecular relapse in the slow responder (2.2% to 9.8% in 157 days), whereas the fast responder had an almost stable allele burden with a slight increase from 1.9% to 4.3% mutated alleles over the course of 462 days (Fig 4B). Interestingly, for the two remaining MR patients, the reduction of the mutant allele burden subsided when the IFN treatment (180 μg/month) entered the maintenance phase (initially 135 μg/month decreasing to 45 μg/month for one patient and 90 μg/month decreasing to 60 μg/month for the other) after which the mutant allele burden remained stable (Fig 4B).

The 17 non-MR patients exhibited relatively stable mutant allele burdens with a median decrease of 2% ranging from a decrease of 45% to an increase of 25%. One PMF patient had a continuous steady decrease in the mutant allele burden of 45% after 700 days of treatment exhibiting the dynamics reminiscent of a MR. However; this was insufficient for achieving MR status at time of data collection (Fig 4A, dashed line).

### Close association between the dynamics of blood cell counts and CALR mutant allele burden

**Fig 5:**
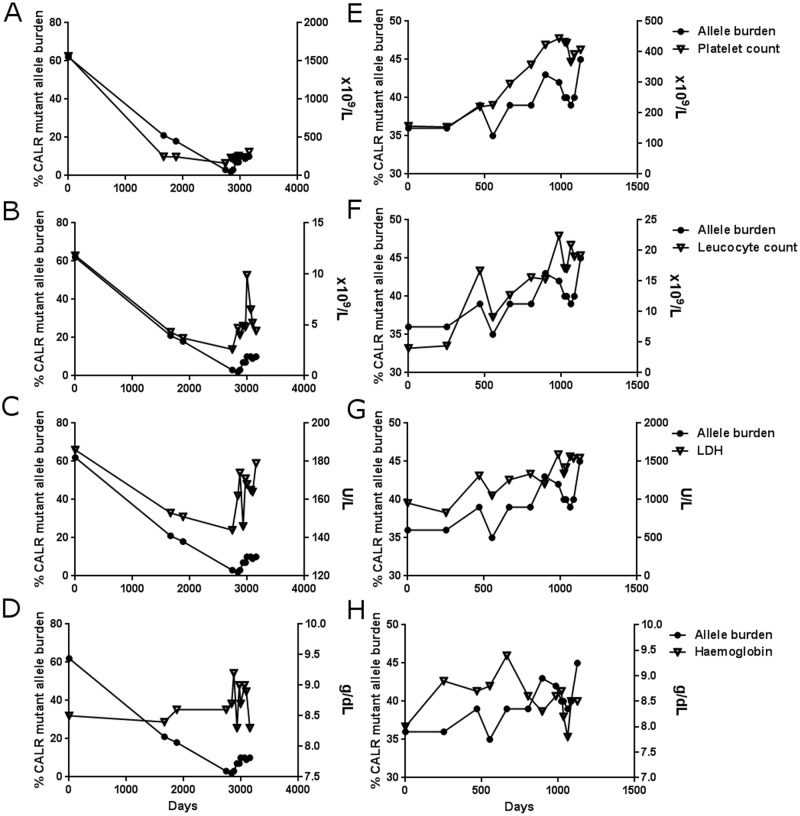
*CALR* mutant allele burden dynamics are closely associated with those of platelets, leucocytes, LDH, and haemoglobin. Representative graphs of the development of the *CALR* mutant allele burden and blood counts for a molecular responder (MR) patient (A-D) and a molecular non-responder (non-MR) patient (E-H) during and after IFN treatment. The values of the mutant allele burdens are shown on the left y-axis. (A and E) Mutant allele burden and platelet count. (B and F) Mutant allele burden and leucocyte count. (C and G) Mutant allele burden and LDH. (D and H) Mutant allele burden and concentration of haemoglobin. Take note of the close resemblance of the dynamics of the mutant allele burden with the blood counts and LDH both decreasing and increasing during relapse (A-D).

To investigate the association of the dynamics of the mutant allele burden with the disease phenotype assessed by standard clinical parameters, we tested all measurements of mutant *CALR* allele burden in the 4 MR paitents and the 14 non-MR patients patients for statistical association with the corresponding leucocyte and platelet counts, plasma LDH, and haemoglobin concentrations during follow-up using a mixed effect model. Irrespective of molecular response and treatment status, the dynamics of the mutant allele burden were positively associated with the platelet count (coeff: 15; 95% CI:[12; 19], P = 2.3x10^-14^) (Fig 5A and 5E), leucocyte count (coeff: 0.13; 95% CI: [0.07; 0.18], P = 7.0x10^-6^) (Fig 5B and 5F), plasma LDH (coeff: 3.1; 95% CI: [1.1; 5.0], P = 0.0023) (Fig 5C and 5G), and negatively with the haemoglobin concentration (coeff: -0.02; 95% CI: [-0.03; -0.01], P = 0.0018) (Fig 5D and 5H).

## Discussion

In the present study, we have developed qPCR assays for monitoring the mutant allele burden of *CALR* type 1 and type 2 mutations. Our qPCR assays demonstrated high sensitivities and reproducibility comparable to those used in routine monitoring of minimal residual disease in MPN patients [31, 36, 37]. Using these assays, we investigated how well the dynamics of the mutant allele burden reflected the disease phenotype during IFN treatment.

Both fragment analysis and Sanger sequencing, in addition to next generation sequencing (NGS) readily detect the wide variety of *CALR* mutations, but do not necessarily reach the recommended assay sensitivity of approximately 1% for molecular diagnosis [38]. The sensitivity of Sanger sequencing is 10–20% and so far, the current NGS techniques are too expensive and time consuming for monitoring allelic burdens and cannot reliably monitor allele burdens below 1–5% in a routine setting making the fragment analysis the method of choice for routine diagnostic screening. However, fragment analysis is unsuited for accurately quantifying the relative abundance of fragments with varying sizes, as it overestimates the amount of the shorter fragment. It is thus suboptimal for estimating the impact of the mutant allele burden on the disease phenotype for *CALR* mutations. Furthermore, the detection limit of the fragment analysis of >1% limits its use for monitoring deep molecular responses. The sensitivity of qPCR assays is dependent on the mutated primer binding specifically to the mutated–and not the wt- sequence. In general, this is only an issue when detecting single nucleotide polymorphisms (SNPs), but the *CALR* exon 9 sequence that harbours the wide variety of *CALR* mutations contains several nucleotide repeats increasing the complexity of assay design. Digital PCR assays circumvent this challenge, as few template copies per reaction chamber ensures virtually no competitive primer binding. Recently, a sensitive assay based on ddPCR for determination of allele burden of type 1 and type 2 *CALR* mutations was published, but so far, few diagnostic laboratories have access to digital PCR and here we present qPCR assays with similar sensitivities [29]. As accurate quantification by qPCR depends on the efficiencies of the assays and is thus vulnerable to batch variations of primers and probes, we ensured accurate qPCR determination by calibrating the assays using reference material obtained by ddPCR. For the type 1 and type 2 mutations our assays thus have utility in being methods available to almost all diagnostic laboratories and in that it increases the sensitivity more than 50 fold compared to available NGS- and fragment analyses. Furthermore, apart from digital PCR it offers the only cost-effective accurate, highly sensitive, and quantitative estimation the type 1 mutation, which is the most common *CALR* mutation.

In our cohort, the type 2 *CALR* mutation was only observed in ET and pre-PMF patients in line with previous reports describing the distribution of *CALR* mutation types [4, 5, 13, 14, 39, 40]. We recorded a slight tendency towards an increased mutant allele burden with a more severe phenotype but could not draw conclusions based on our small groups. It has previously been suggested that the mutant allele burden of *CALR* increases with a more severe phenotype as also reported for *JAK2-*V617F [5, 41]. However, it should be kept in mind that the fragment analysis induces overestimation of type 1 mutations predominantly found in PMF, thus biasing this observation.

Since Verger *et al*., have demonstrated that hydroxyurea and anagrelide do not have an impact on the mutant allele burden in *CALR* mutated patients, we do not consider these treatments to be an issue for evaluating the effects of IFN in reducing the mutant allele burden.

In the current study we adopted the ELN 2009 guidelines, which is normally used to assess the molecular response of the *JAK2*-V617F mutation. However, the the study by Verger *et al*. also included patients with reductions in allele burden ranging from 25% to 50%, thus increasing the number of molecular responders. In our study, two patients designated as non-MR patients were in this category [22]. Currently, clinical value and prognostic significance of the different levels of reduction in *CALR* mutant allele burden as well as its rate of reduction is unclear and need to be investigated for larger cohorts. Here, we extend previous findings by demonstrating molecular responses in patients with pre-PMF and PMF with high and intermediate initial mutant allele burdens indicating that IFN may induce molecular remissions regardless of the MPN diagnosis of *CALR* mutated patients and their initial tumor load [22].

Our study revealed substantial variations in IFN exposure times required to obtain a molecular response suggesting that a subset of patients exhibit particularly slow elimination kinetics. In addition, considering LDH as a marker for malignant cell turnover, among the non-MR patients, we found two patients that did not obtain normalisation of LDH until 2 years after achieving haematological remission, suggesting even slower elimination kinetics. Quintás-Cardama and co-workers stated that significant molecular responses demanded at least 6 months of IFN treatment, influencing our initial inclusion criteria [42]. However, our data emphasise that a subset of *CALR* mutated patients demands much longer IFN exposures to substantiate a molecular response.

Two patients achieved a sustained complete haematological response more than a year after discontinuation of IFN treatment suggesting induction of long-term treatment-free haematological responses in *CALR* mutated patients. It is noteworthy that the patient with a rapid initial reduction in mutant allele burden only had a slight increase in the mutant allele burden after withdrawal of IFN, whereas the patient with particularly slow reduction experienced a very rapid molecular relapse upon discontinuation of IFN. The initial elimination kinetics for the *CALR* mutant allele burden may thus hold a prognostic value for the duration of treatment free remissions, and the mutant allele burden appears to be a more sensitive marker of relapse than standard blood cell counts.

The loss of further reduction in the mutant allele burden in the MR patients when entering maintenance phase of IFN treatment indicates not surprisingly that a certain level of IFN is required for reduction of the malignant clone. This may also explain the discrepancy in molecular response observed in the current study (>50% reduction in mutant allele burden in 20% of patients and 50% > x >25% reduction in 10% of patients) and the study by Verger *et al* (>50% reduction in mutant allele burden in 43% of patients and 50% > x >25% reduction in 20% of patients) as higher IFN concentrations were used in the French study [22, 23].. Accordingly, *CALR* positive patients may require higher IFN-dosages to achieve major molecular remissions contrasting the low-dose IFN treatment options used to achieve molecular remissions in half of *JAK2-*V617F positive ET and PV patients. None of our patients experienced a reduction in mutant allele burden below 1%, which, in part, may be due to discontinuation or lowering of the IFN dose. However, it should be noted that in our study, the term “maintenance phase” covers a necessary lowering of dosage due to adverse effects. Moreover, considering the somewhat more aggressive treatment regime used by Verger *et al* it is interesting that the toxicity based dropout rates between the studies are comparable (24% versus 19%).

Neither the mutant allele burden, diagnosis nor haematological parameters predicted molecular response. However, we noted that absence of molecular response could implicate a more advanced disease stage assessed by anaemia, bone marrow fibrosis, and palpable splenomegaly.

A predictive factor for obtaining a molecular response to IFN treatment in *CALR* mutation positive patients has eluded researchers, but additional mutations appear to be associated with a suboptimal response [22, 42], and we are currently undertaking investigations of the mutational status of the IFN treated patients to examine prognostic markers for the differential dynamics in the MR group.

Similar to observations of the mutant allele burden in *JAK2-*V617F mutated ET patients, where the mutant allele burden correlates with leucocyte–and platelet count, and plasma LDH [41, 43], studies have indicated that higher mutant allele burdens for *CALR* mutated patients may also correlate with increased leucocyte- and platelet counts, and with a lower haemoglobin concentration at the time of diagnosis [5, 14]. In the current study, we show a highly significant association between the dynamics of the mutant allele burden and the haematological parameters during and after IFN treatment. The dynamics of the mutant allele burden are closely associated with fluctuations in leucocyte- and platelet counts; in addition to plasma LDH levels, as well as inversely associated with haemoglobin concentration. The *CALR* mutations are considered an initiating event during the development of ET and PMF patients [2, 3, 44]. In agreement with this hypothesis, the close similarities of the dynamics of the mutant allele burden and haematological parameters we herein report support the contention that the mutant *CALR* allele load is a direct measure and determinant of “disease burden”.

Presently, no uniform international agreement exists regarding indications for discontinuation of IFN treatment in MPN. The Danish national guidelines for treatment of ET allows discontinuation of IFN in *JAK2*-V617F positive patients with a sustained reduction of mutant allele burden below 1%. None of our patients in this study achieved such a deep remission and in the light of the two patients with molecular relapse after IFN withdrawal, we suggest a similar approach to *CALR* mutation positive patients.

In conclusion, we have developed a sensitive and robust qPCR assay for determination of the mutant allele burden in the two most common *CALR* mutations. We expand previous findings of IFN efficacy in ET patients by showing molecular response in pre-PMF and PMF patients as well. Importantly, we have demonstrated that variation in the dynamics of mutant *CALR* allele burden during and after treatment is closely associated with haematological parameters and thus likely reflects the disease burden. We suggest performing robust and routine accessible *CALR* qPCR based monitoring of *CALR* type 1 and 2 mutated patients in all future MPN trials.

## Supporting Information

S1 Fig Design considerations for the type 1 design. Sequences for wildtype (wt) and type 1 mutation where the seven base pair (bp) repeats flanking the deletion is underlined in the wt sequence. The 7 bp repeat at the 3’ end of the deletion is shown in blue. The yellow box shows the wt downstream part of the deleted sequence which is to be covered by one end of the specific primer. The other end overlaps the part upstream of the deletion and the primer specificity is dependent on differences between the mutated and the wt sequence. Differences are shown by red colouring of bases and the strength of the mismatch is indicated below, where S is a strong mismatch, M is a medium mismatch and W is weak. The common seven bp repeat and the fact that most mismatches of the specific part of the primer are weak complicates the design.
(TIF)
