# Supplementary figures and images for: Differential Dynamics of CALR Mutant Allele Burden in Myeloproliferative Neoplasms during Interferon Alfa Treatment
Source: PLoS One. 2016 Oct 20;11(10):e0165336. doi: 10.1371/journal.pone.0165336 (PMC5072743; doi:10.1371/journal.pone.0165336)

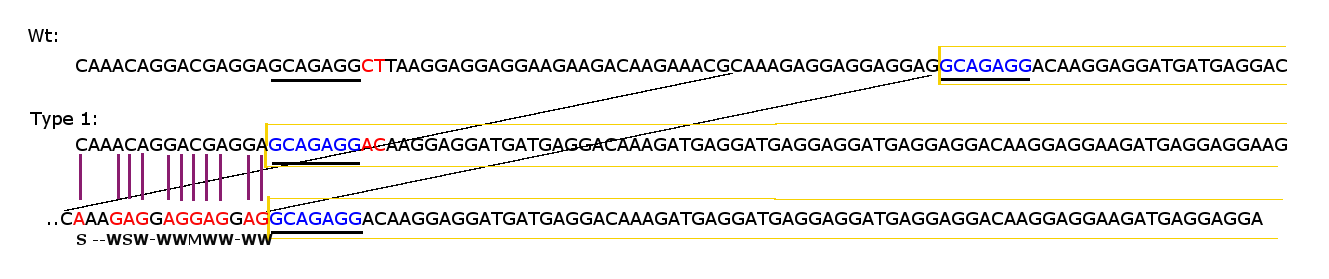

Supplement: S1 Fig — Sequences for wildtype (wt) and type 1 mutation where the seven base pair (bp) repeats flanking the deletion is underlined in the wt sequence. The 7 bp repeat at the 3’ end of the deletion is shown in blue. The yellow box shows the wt downstream part of the deleted sequence which is to be covered by one end of the specific primer. The other end overlaps the part upstream of the deletion and the primer specificity is dependent on differences between the mutated and the wt sequence. Differences are shown by red colouring of bases and the strength of the mismatch is indicated below, where S is a strong mismatch, M is a medium mismatch and W is weak. The common seven bp repeat and the fact that most mismatches of the specific part of the primer are weak complicates the design. (TIF) [file pone.0165336.s001.tif]
